# Overcoming IGF1R-Mediated Resistance to Oncolytic HSV1 and Radiotherapy via Triple Combination Therapy

**DOI:** 10.21203/rs.3.rs-8117591/v1

**Published:** 2025-12-15

**Authors:** Ji Young Yoo, Alexandra Miller, Minhye Noh, Jin Muk Kang, Amanda Kouaho, Grace Nguyen, Minxin Huang, Stephanie Bean, Sunil Krishnan, Zhongming Zhao, E. Antonio Chiocca, Tae Jin Lee, Jiyeon Kim

**Affiliations:** University of Texas Health Science Center at Houston; University of Texas Health Science Center at Houston; University of Texas Health Science Center at Houston; University of Texas Health Science Center; University of Texas Health Science Center at Houston; The University of Texas; Stony Brook University Graduate School; University of Texas Health Science Center at Houston; The University of Texas Health Science Center at Houston; The University of Texas Health Science Center at Houston; Brigham and Women’s Hospital; University of Texas Health Science Center at Houston; University of Texas Health Science Center at Houston

**Keywords:** Oncolytic herpes simplex virus-1 (oHSV), Glioblastoma (GBM), Breast Cancer (BC), Insulin-like growth factor-1 receptor (IGF1R), Tumor microenvironment (TME), Therapeutic Synergy, Therapeutic resistance

## Abstract

FDA-approved oncolytic herpes simplex virus-1 (oHSV) therapy has emerged as an effective viro-immunotherapy for solid tumors. However, tumor- and tumor microenvironment (TME)-associated adaptations following viral treatment, such as feedback immune suppression, neoangiogenesis, and enhanced tumor aggressiveness, often hinder complete tumor eradication. Gaining a deeper understanding of the molecular mechanisms that limit the therapeutic efficacy of oHSV will be crucial to enhancing its clinical impact. We recently discovered that oHSV induces Insulin-like growth factor 2 (IGF2) secretion, shaping an immunosuppressive TME. Similarly, radiotherapy (RTx) activates the IGF1/IGF1R and YAP1 signaling pathways, further promoting therapeutic resistance. In this study, we investigated how oHSV-induced IGF1R/YAP1 signaling influences feedback pro-survival and proliferative pathways in tumor cells and evaluated the therapeutic potential of combining IGF1R blockade with oHSV and RTx.

We first demonstrated that oHSV activates IGF1R signaling *in vitro* and *in vivo*, promoting tumor proliferation. While IGF1R-targeted monotherapies showed limited cytotoxic effects, combining IGF1R inhibitors with oHSV led to a significant, albeit modest, increase in cytotoxicity across tested *in vitro* breast cancer (BC) and primary glioblastoma (GBM) cells and *in vivo* xenograft models. Furthermore, we observed that co-treatment with oHSV and RTx robustly activated both IGF1R and YAP1 in resistant cells, revealing the IGF1R/YAP1 axis as a key mediator of resistance to dual oHSV and RTx therapy. Notably, the triple combination of oHSV, RTx, and IGF1R blockade yielded synergistic anti-tumor effects, abolished YAP1 expression, and significantly enhanced survival in orthotopic BC and GBM models. Collectively, these findings provide a strong rationale for the clinical evaluation of triple-combination therapy as a synergistic strategy to enhance the anti-tumor efficacy of oHSV and overcome RTx resistance in patients with BC and GBM.

## Introduction

Oncolytic herpes simplex virus-1 (oHSV) is FDA-approved for melanoma^1, 2^ and conditionally approved for glioblastoma (GBM); however, clinical responses remain limited.^3^ Resistance arises from both tumor-intrinsic and -extrinsic mechanisms, including dysregulated interferon signaling, aberrant growth factor pathways, and pronounced tumor heterogeneity.^4, 5^ Moreover, oHSV can paradoxically suppress anti-tumor immunity and activate pro-survival pathways such as PI3K/AKT and mTORC1, promoting tumor persistence.^6, 7^

Our group recently discovered that oHSV infection stimulates insulin-like growth factor 2 (IGF2) secretion, which in turn activates the insulin-like growth factor 1 receptor (IGF1R)-AKT signaling throughout the tumor microenvironment (TME). This activation fosters immune suppression and diminishes the therapeutic efficacy of oHSV.^8^ In addition, viral proteins such as Us3 and virus-induced host factors such as heparinase further augment these pro-survival signaling cascades.^9, 10^ Together, these findings underscore a critical therapeutic challenge: how to preserve and enhance the immunogenic and oncolytic functions of oHSV while mitigating virus-induced, tumor-supportive signaling.

The insulin-like growth factor (IGF) system, a complex autocrine and paracrine network composed of ligands (IGF1, IGF2), receptors (IGFIR, IGF2R), and six IGF-binding proteins (IGFBP1 to IGFBP6), is frequently overexpressed in cancer and is associated with poor prognosis in GBM and breast cancer (BC).^11–15^ IGF1R signaling promotes metastasis and pre-metastatic niche formation.^16, 17^ It is also implicated in driving resistance to radiation therapy (RTx), temozolomide (TMZ), and receptor tyrosine kinase (RTK) inhibitors in GBM and BC.^11, 18–21^ Specifically, IGF1R activation following RTx and TMZ, standard of care (SOC) for intracranial malignancy, induces autophagy, stemness, senescence, and DNA repair, thereby attenuating treatment-induced apoptosis.^22, 23^ Repeated RTx upregulates IGF1R expression and IGF1 secretion, further enhancing PI3K/Akt signaling and N-cadherin expression, which promotes tumor stemness and regrowth.^19, 24^ Despite extensive interest in therapeutically targeting IGF1R signaling in GBM and BC, clinical inhibitors have shown limited success, likely due to compensatory activation of redundant RTKs such as EGFRwt, EGFRvIII, PDGFR, FGFR, and HER2.^11, 13, 25^

Herein, we expand upon our prior work demonstrating that oHSV-induced IGF2/IGF1R signaling promotes immunosuppression to explore its impact on cancer cell survival and resistance. We found that enhanced IGF1R activation occurs exclusively near sites of active viral replication, promoting tumor proliferation. While combining oHSV with IGF1R inhibition modestly enhanced therapeutic efficacy, the required doses impeded viral replication. Similarly, dual oHSV and RTx therapy showed limited benefit, further increasing IGF1R activation and inducing dose-dependent expression of the oncogenic and immunosuppressive factor YAP1 in resistant cells. Importantly, this IGF1R activation sensitized GBM and BC tumors to IGF1R-targeted therapy. Triple combination therapy with oHSV, RTx, and IGF1R blockade synergistically improved survival, abolished YAP1 expression, and reduced cancer cell self-renewal. Together, these findings identify IGF1R signaling as a key driver of resistance to oHSV therapy and support triple-combination strategies as a promising approach for the treatment of GBM and BC.

## Materials and Methods

### Ethics Statement

All mouse housing and experiments were performed in accordance with the Animal Welfare Committee (AWC) at the University of Texas Health Science Center in Houston guidelines and have been approved by the Institutional Review Board.

### Cell lines and Oncolytic Herpes Simples Virus-1 (oHSV-1)

All cell lines, primary GBM cells, and viruses used in this study are described in the **Supplementary Materials and Methods**.

Cell Proliferation Assay, Western Blotting, Immunohistochemistry, Immunofluorescence, Flow Cytometry, Soft Agar Colony Forming Assay, and qRT-PCR

All commercial kits, and antibodies (**Supplementary Table S2**) are provided in the supporting data. Original full-length images of western blotting analysis are provided (Supplemental WB Proofs). Detailed descriptions of these methods can be found in the **Supplementary Materials and Methods**.

### Animal Studies

Six- to eight-week old NSG, athymic nu/nu, and C57BL/6 mice were obtained from Jackson Laboratory (Bar Harbor, ME). All details of orthotopic stereotactic intracranial tumor, subcutaneous tumor, and orthotopic mammary fatpad implantation and treatment are described in the **Supplementary Materials and Methods**.

### Statistical Analysis

Statistical analyses were performed using GraphPad Prism version 10 (GraphPad, San Diego, CA, USA RRID:SCR_002798)) or Python (Version 3.13). Unpaired student’s t-test was used to test continuous data with false discovery test described in the **Supplementary Materials and Methods**. To analyze survival data, Kaplan-Meier curves were compared using the log-rank test and the post hoc pairwise groups test was performed by Benjamini and Hochberg correction. Dose-response curves, synergy and sensitivity^26^ calculations were made using SynergyFinder + and summarized in **Supplementary Table S1**.^27^ All statistics are summarized in the **Supplemental Statistics** and the data files for upload to SynergyFinder + are available in the **Supplemental Synergy Data**.

## Results

### oHSV infection promotes tumor proliferation through MAPK and PI3K-AKT signaling in GBM and breast cancer brain metastasis (BCBM).

**Figure 1:**
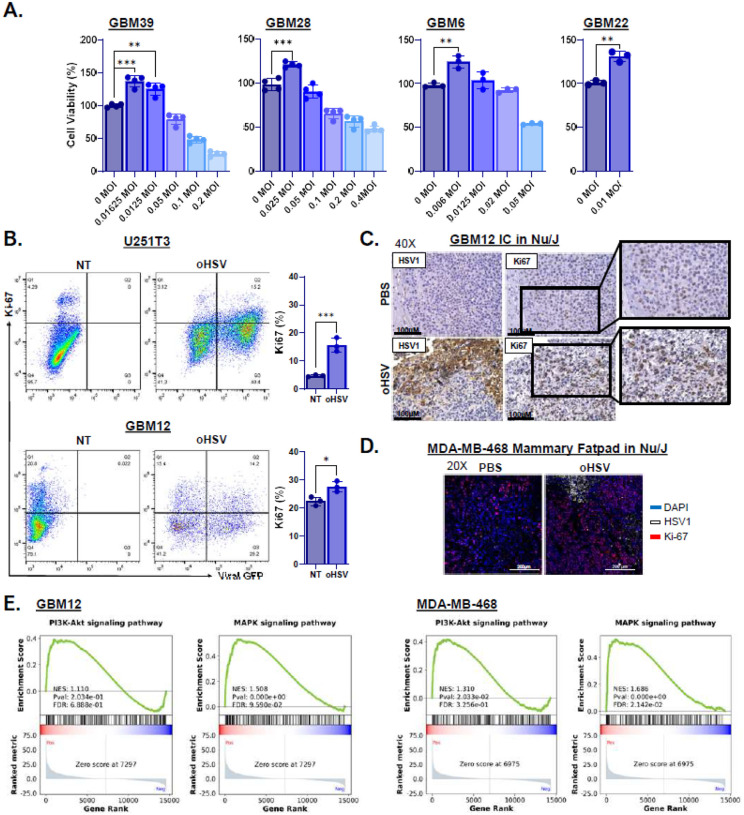
oHSV infection promotes proliferation via activation of MAPK and PI3K-AKT signaling in GBM and BCBM. (A) Standard MTT Cell Viability assay of patient-derived GBM cells treated with or without oHSV at various MOI for 96 hours (*n* = 4/group). Cell viability was determined relative to the untreated samples. (B) (left) Representative dot plots of Ki-67 flow cytometry of the U251T3 glioma cell line (MOI = 0.1) and patient-derived primary GBM12 cells (MOI = 0.05) treated with or without oHSV for 24hours. Total percentage of +Ki-67-positive cells performed in triplicate is depicted as bar graph (right). (C) Representative histological analysis of oHSV-induced proliferation measured by Ki-67 staining in intracranial GBM12 tumor-bearing brain section from mice treated with or without oHSV (Magnification, 40X). (D) Representative fluorescent microscopy images stained for HSV1 (white), Ki-67 (red), and counterstained with DAPI (blue) (Magnification 20X). (E) Gene set enrichment (GSEA) plots of PI3K-AKT and MAPK signaling in GBM12 and MDA-MB-468 BC cells (n = 4/group) treated with or without oHSV (MOI 0.1) for 16 hours, analyzed using previously published mRNA-sequencing data^8^. All data are presented as means ± SEM with * p < 0.01. ** p < 0.005. *** p < 0.0005. ns = not significant. All unspecified interactions were not significant.

We previously showed that IGF2 secreted from oHSV-infected tumor cells reduces therapeutic efficacy and that targeting IGF2 can reprogram the TME to enhance viro-immunotherapy. Here, we investigated whether oHSV-induced IGF1R signaling contributes to therapeutic resistance and whether its inhibition could improve oHSV efficacy. We used rHSVQ, an oHSV with γ34.5 deletion and ICP6 disruption, which shares a backbone with clinical candidates CAN-3110 (NCT03152318) and G207 (NCT00028158). High-dose oHSV exhibits robust cytotoxicity, but low-dose infection paradoxically enhanced cell proliferation, especially in resistant GBM cells (GBM6, GBM22, and GBM39) (Fig. 1A). Flow cytometry confirmed increased Ki67 expression in subsets of surviving infected and uninfected cells (Fig. 1B, **Fig. S1**). *In vivo*, oHSV treatment increased Ki67 staining in GBM12 and orthotopic MDA-MB-468 BC tumors, with Ki67-positive cells exclusively localized near regions of viral replication (Fig. 1C–D, **Fig. S2–3**). Gene set enrichment analysis (GSEA) of our previously published RNA sequencing (RNA-Seq) data revealed significant activation of MAPK, PI3K-Akt, and FoxO pathways in oHSV-infected GBM12 and MDA-MB-468 cells (Fig. 1E, **Fig. S4A-B**), suggesting that while HSV-1 exploits these cascades for replication, they also drive tumor regrowth and immune evasion.^8^

### oHSV infection induces IGF1R signaling in GBM and BCBM

**Figure 2:**
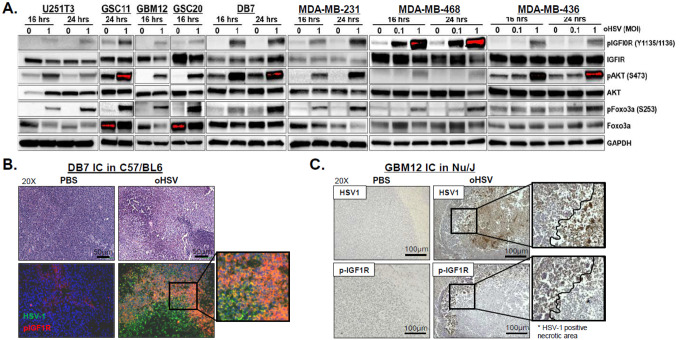
oHSV infection induces activation of IGF1R signaling in GBM and BCBM. (A) Western blot analysis of pathways downstream of IGF1R in patient-derived primary GBM cells and BC cell lines treated with or without oHSV (MOI = 0.1, 1) for 16 or 24 hours. (B) H&E staining (top) and representative merged fluorescence microscopy images showing oHSV-induced activation of IGF1R signaling in intracranial GBM12 tumor-bearing brain sections from mice treated intratumorally with or without oHSV (5×10^5^ pfu) (bottom). HSV-1 (green) and p-IGF1R (Y1135/1136) (red) were visualized, with nuclei counterstained with DAPI (blue). (C) Histological analysis of oHSV-induced IGF1R activation in intracranial GBM12 tumor-bearing brain sections treated with or without oHSV. (Magnification, 20X for all microscopy).

While we previously demonstrated that IGF2 contributes to immune evasion,^8^ the impact of oHSV-induced IGF1R activation on tumor cell signaling remains unclear. Given that the MAPK, PI3K-Akt, and FoxO signaling cascades act downstream of IGF1R, we investigated their activation by western blot (Fig. 2A). Consistent with RNA-Seq data, oHSV induced dose- and time-dependent increases in phosphorylation of IGF1R and Akt (S473) in BC and glioma cell lines, and patient-derived primary GBM cells. Consistent with RNA-seq data, oHSV treatment induced dose- and time-dependent phosphorylation of IGF1R and Akt (S473). Subsequent phosphorylation of FoxO3a (S253) promoted its cytoplasmic sequestration and proteasomal degradation, evidenced by reduced total FoxO3a protein, likely contributing to post-treatment proliferation (Fig. 2A).^28^ Similarly, histology and immunofluorescence (IF) of orthotopic GBM12 and DB7 murine BCBM tumors further confirmed enhanced IGF1R phosphorylation in and around regions of viral replication (Fig. 2B–C, **Fig. S5–6**). Notably, IGF1R activation persisted within both virus-infected and surrounding tumor areas despite extensive necrosis. Collectively, these findings suggest that oHSV-induced proliferation is largely driven by IGF1R signaling, potentially sensitizing tumors to IGF1R-targeted therapies.

### oHSV treatment sensitizes infected primary GBM and BCBM to IGF1R inhibition, enhancing anti-tumor efficacy.

**Figure 3:**
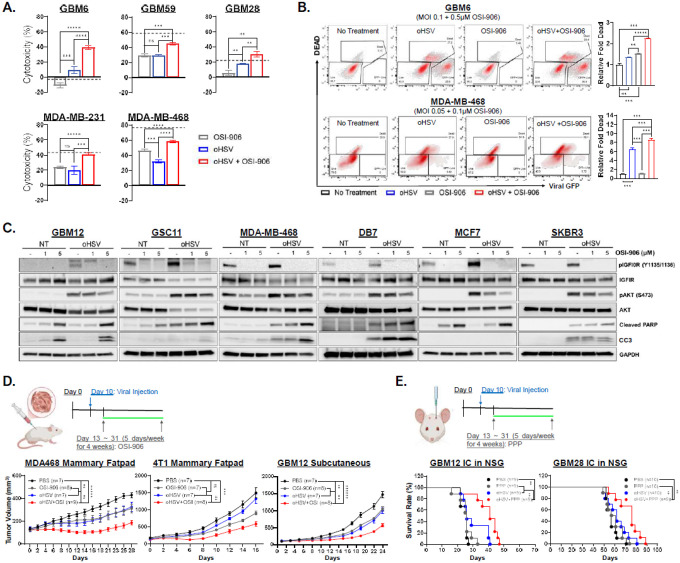
oHSV treatment sensitizes infected primary GBM and BCBM cells to IGF1R inhibition, enhancing anti-tumor efficacy. (A) MTT Cell Viability assay of patient-derived primary GBM and BC cells treated with or without oHSV and incubated for two hours then treated with DMSO solvent control or the small molecule IGF1R-inhibitor, OSI-906 (0.05, 0.1, 0.5, 1, or 5μM) for 96 hours *(n* = 4/group).Cytotoxicity was determined relative to the untreated samples. (B) Flow cytometry of Live/Dead staining and virally expressed GFP of GBM6 and MDA-MB-468 was performed 48 hours post-treatment with or without oHSV (MOI = 0.5 and 0.2, respectively) and with or without OSI-906 (0.1 or 0.5 μM OSI-906 respectively). The relative fold change in the percentage of dead cells was determined relative to the untreated/DMSO control group *(n* = 3/group). (C) Western blot analysis of patient-derived primary GBM12 and GSC11 cells treated with or without oHSV (MOI = 0.5 and 0.2, respectively) and BC cells treated with or without oHSV (MOI = 0.5) for 2 hours then either DMSO, 1, or 5 μM of OSI-906 for 18 hours. (D) Patient-derived GBM12 cells were implanted subcutaneously, and BC cells (MDA-MB-468 and 4T1) were implanted into the mammary fatpad of athymic nude mice, which were then treated with PBS or oHSV intratumorally on days 10 and 13. Mice were then treated with 25mg/kg OSI-906 5, days per week for 4 weeks and tumor volume was monitored. Group sizes are indicated in the figures. (E) Kaplan-Meir survival curve of mice bearing intracranial GBM12 or GBM28 tumors treated intratumorally with PBS or oHSV 10 days after implantation then treated with solvent control or 25mg/kg PPP by oral gavage 5 days per week for 4 weeks. All data are presented as means ± SEM with * p < 0.01. ** p < 0.005. *** p < 0.0005, **** p < 0.00005, ***** p < 0.000005. ns = not significant.

To evaluate whether oHSV treatment sensitizes cancer cells to IGF1R inhibition, we used OSI-906, a potent orally available small-molecule IGF1R inhibitor. Cells were infected with rHSVQ for 1 hour and treated with OSI-906 (0.05–5μM) or DMSO. A standard MTT assay at 96 hours showed that combination treatment modestly increased cancer cell killing compared to either monotherapy but was largely additive (dotted line) rather than synergistic (Fig. 3A). Live/dead cell staining for flow cytometry further confirmed an additive increase in MDA-MB-468 and GBM6 (Fig. 3B, **Fig. S7**). Western blot revealed effective inhibition of IGF1R activation and dose-dependent increase in cleaved PARP and cleaved caspase 3 (CC3), indicating enhanced apoptotic cell death (Fig. 3C). However, OSI-906 also significantly impaired oHSV replication and propagation in a dose-dependent manner at OSI-906 concentrations ≥ 0.5μM, as shown by viral GFP quantification using Cytation 5 Live Cell Imaging and plaque-forming assays (**Fig. S8–9**).

*In vivo*, combination therapy significantly reduced tumor burden in orthotopic MDA-MB-468 and 4T1 BC models more effectively than monotherapy (Fig. 3D, **Supplemental Statistics**). In contrast, OSI-906 showed no benefit in intracranial GBM12 and GBM28 models due to poor blood-brain barrier (BBB) penetration (**Fig. S10**). Using picropodophyllin (PPP), a BBB-permeable IGF1R inhibitor (**Fig. S11**), we observed significantly prolonged survival with combination therapy: median survival increased from 26 to 41 days in GBM12 and from 62 to 76 days in GBM28 (*P* = 0.0109 and 0.0347, respectively) (Fig. 3E, **Supplemental Statistics**). Collectively, these results suggest that IGF1R blockade enhances oHSV efficacy but remains insufficient for tumor eradication.

### Combined oHSV and RTx enhances IGF1R activation, promoting YAP1 expression.

**Figure 4:**
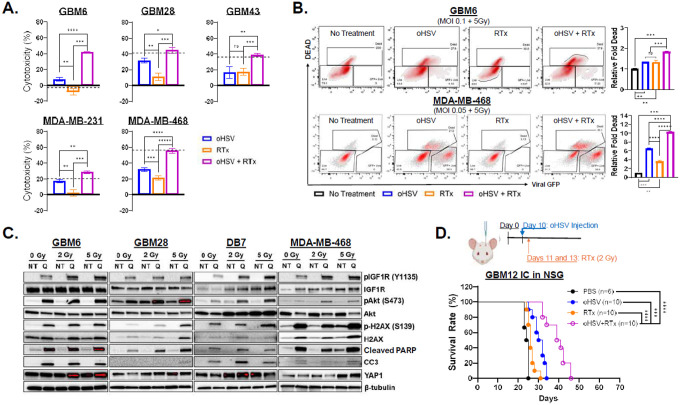
Combined oHSV and radiotherapy (RTx) enhances IGF1R activation in a dose-dependent manner, promoting YAP1 expression. (A) MTT Cell Viability assay of patient-derived primary GBM and BC cells treated with or without oHSV and incubated for two hours then treated with or without RTx (2 or 5 Gy) for 96 hours *(n* = 4/group). Cytotoxicity was determined relative to the untreated samples. (B) Flow cytometry of Live/Dead staining and virally expressed GFP of GBM6 and MDA-MB-468 was performed 48 hours post-treatment with or without oHSV (MOI = 0.5 and 0.2, respectively) and with or without RTx (5Gy). The relative fold change in thepercentage of dead cells was determined relative to the untreated/DMSO control group *(n* = 3/group). (C) Western Blot analysis of patient-derived GBM cells and BC cells treated with or without oHSV (MOI = 0.2) then 1 hour later treated with or without radiotherapy (2, 5 Gy). (D) Kaplan-Meir survival curve of intracranial GBM12 tumor-bearing mice treated intratumorally with PBS or oHSV 10 days after implantation then treated with or without 2 Gy of RTx on days 11 and 13. All data are presented as means ± SEM with * p < 0.01. ** p < 0.005. *** p < 0.0005, **** p < 0.00005. ns = not significant. All unspecified interactions were not significant.

Radiotherapy (RTx) remains a cornerstone of the standard-of-care (SOC) for GBM and BCBM but induces IGF1/IGF1R signaling, triggering radio-resistance and recurrence.^24^ As expected, combining oHSV with RTx significantly increased cytotoxicity across GBM and BC cells, either additively or synergistically, as measured by MTT assay (Fig. 4A, **Supplemental Statistics**). Live/dead cell staining confirmed additive enhancement of cell death (Fig. 4B, **Fig. S7**). Western blot revealed marked increases in γH2AX, cleaved PARP, and CC3, indicating enhanced DNA damage response and apoptotic cell death (Fig. 4C). Combination therapy further activated IGF1R-Akt signaling in an RTx dose-dependent manner (Fig. 4C).

RTx is known to induce YAP1 expression, a transcription factor that promotes radio-resistance, stemness, and immunosuppression.^29–31^ Consistent with this, we observed RTx dose-dependent upregulation of YAP1 and IGF1R, particularly in GBM6 and DB7, oHSV resistant cells (Fig. 3C). Kaplan-Meier analysis of GBM12 tumor-bearing mice showed improved survival with combination therapy (median survival: oHSV = 31 days; RTx = 26 days; combination = 40 days, *P < 0.001* vs. oHSV, *P < 0.0001* vs. RTx) (Fig. 4D). Collectively, these findings demonstrate that while oHSV + RTx enhances therapeutic efficacy, it simultaneously promotes IGF1R/YAP1 signaling, which may drive residual resistance.

### Combined RTx and oHSV treatment synergizes with IGF1R inhibition in vitro and in vivo.

**Figure 5:**
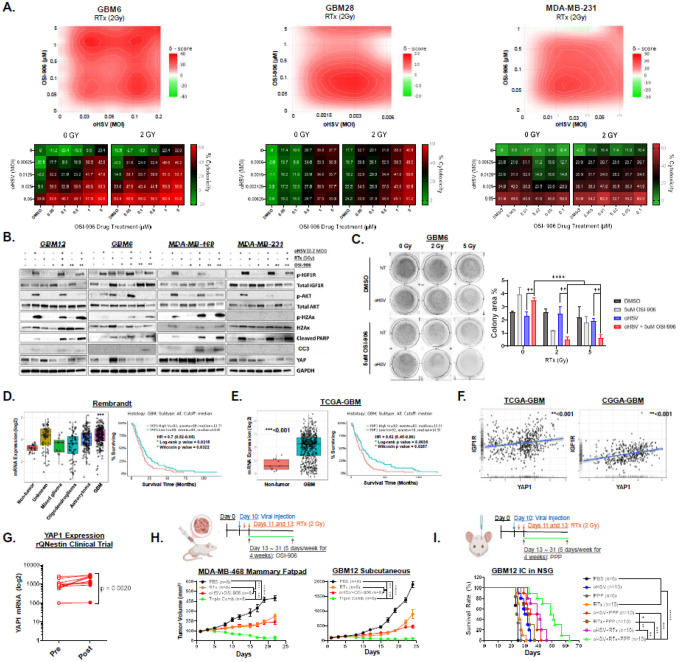
Combined RTx and oHSV treatment synergizes with IGF1R inhibition *in vitro* and *in vivo*, limiting self-renewal capacity. (A) MTT Cell Viability assay of patient-derived primary GBM and BC cells treated with or without oHSV, then with or without RTx (2, 5 Gy), then 1 hour later treated with DMSO solvent control or OSI-906 (0.05, 0.1, 0.5, 1, or 5μM) for 96 hours *(n* = 4/group). Bliss synergy interaction landscapes at 2Gy are depicted (top) along with heatmaps of cytotoxicity relative to no treatment. (B) Western blot analysis of patient-derived primary GBM cells and BC cell lines treated with triple combination therapy or monotherapy as in A. (C) Representative wells of soft agar colony-forming assay of GBM6 cells treated as in A (left) and total area covered by colonies quantified as a percentage of total area using the ColonyArea plugin for FIJI^63^. (D) The log2 mRNA expression of YAP1 in gliomas (left) and Kaplan-Meir survival curve with median split grouping based on YAP1 expression (right) acquired using the Rembrandt data set. (E) The log2 mRNA expression of YAP1 in GBM (left) and Kaplan-Meir survival curve with median split grouping based on YAP1 expression (right) in female patients acquired using the Agilent-4502A Platform TCGA-GBM data set. (F) Correlation between log2 mRNA expression of IGF1R and YAP1 acquired using the TCGA-GBM (left) and CGGA-GBM (right) data sets. (G) YAP1 gene expression levels pre- and post-rQNestin34.5v2 treatment in 14 recurrent GBM patients (ClinicalTrials.gov, NCT03152318). (H) Patient-derived GBM12 cells were implanted subcutaneously and MDA-MB-468 cells were implanted into the mammary fatpad of athymic nude mice then treated with PBS or oHSV intratumorally on day 10 and 13. Mice were then treated with or without RTx (**2 Gy**) followed by 25mg/kg OSI-906 5 days per week for 4 weeks and tumor volume was measured (mm^3^). Group sizes are shown in the figure. (I) Kaplan-Meir survival curve of intracranial GBM12 tumor-bearing mice treated intratumorally with PBS or oHSV 10 days after implantation then with or without RTx (**2** Gy), then treated with solvent control or 25mg/kg PPP by oral gavage 5 days per week for 4 weeks. All data are presented as means ± SEM with * p < 0.01. ** p < 0.005. *** p < 0.0005, **** p < 0.00005, ***** p < 0.000005. ns = not significant. All unspecified interactions were not significant.

We next investigated whether RTx- and oHSV-induced activation of IGF1R signaling sensitizes tumor cells to IGF1R-targeted therapy. Cells were infected with oHSV (0.001–0.2 MOI), treated one hour later with RTx (0 or 2 Gy), and then exposed to OSI-906 (0.05–5μg/ml). Cytotoxicity measured by MTT assay at 96 hours revealed strong synergy with triple combination in patient-derived GBM cells (GBM6, GBM28) and BC cells (MDA-MB-231), as measured by SynergyFinder + analysis (Fig. 5A, **Fig. S12, Table S1**)^32^. Similarly, the combined sensitivity score (CSS) and relevant inhibition (RI) scored confirm that dual oHSV and RTx therapy sensitize tumors to IGF1R inhibition (**Table S1**). Notably, while Bliss synergy scores indicated overall antagonism in MDA-MB-231 (mean = −3.74, *P* = 3.56e-3) at higher OSI-906 doses, marked synergy was observed at lower dosage (<μM concentrations), particularly at lower oHSV and RTx doses. The Loewe model confirmed strong synergy (mean = 15.12, *P* = 1.77e-18), while dual therapy combinations showed antagonistic interactions not present with the triple combination (**Fig. S12B**).

Mechanistically, triple combination therapy reduced IGF1R-Akt activation, enhanced DNA-damage responses, and increased PARP and Caspase-3 cleavage, with a consistent dose-dependent decrease in YAP1 expression across all cell types tested (Fig. 5B). Functional assays further demonstrated markedly reduced colony formation and proliferation (Fig. 5C, **Fig. S13**).

Analysis of the Rembrandt and TCGA databases supported these findings. YAP1 expression was elevated in GBM versus non-tumor brain tissue (Fig. 5D–E, left) and negatively correlated with patient survival, particularly in female patients (Fig. 5D–E, right **Fig. S14**). Furthermore, YAP1 positively correlated with IGF1R gene expression in GBM in both the TCGA and Chinese Glioma Genome Atlas (CGGA) datasets (Fig. 5F). Importantly, tumor samples from 12 recurrent GBM patients treated with rQNestin34.5v.2 (ClinicalTrials.gov: NCT03152318) showed increased YAP1 expression in 7 patients (58.33%; *P* = 0.0020), underscoring the clinical relevance of YAP1 upregulation in response to oHSV therapy (Fig. 5G).^33^ YAP1 expression is similarly deleterious in BCBM, where enhanced expression increases the probability of brain metastasis in the TCGA-BRCA cohort (**Fig. S15**).

Finally, the triple combination therapy markedly reduced tumor burden in orthotopic MDA-MB-468 xenografts, with mean tumor volumes by day 22 of 432.21 ± 27.3 mm³ in controls, 248.4 ± 23.3 mm³ with RTx (42.5% inhibition, P < 0.001), 183.8 ± 20.7 mm³ with oHSV + OSI-906 (57.4% inhibition, P < 0.001), and 49.6 ± 23.3 mm³ with the triple combination therapy (88.5% inhibition, P < 0.000001), and complete tumor regression in 5 out of 8 mice (Fig. 5H left). Similar results were seen in subcutaneous GBM12 models, including complete tumor regression in 2 out of 8 mice (P < 0.001 vs RTx or oHSV + OSI-906) (Fig. 5H right). Kaplan-Meier survival analysis further demonstrated that the triple combination therapy significantly extended survival in intracranial GBM12-bearing mice (median = 50 days) compared to dual therapies (P < 0.0001 vs. dual therapies; median survival: oHSV + PPP = 36.5 days; oHSV + RTx = 40 days; or RTx + PPP = 32.5 days) (Fig. 5I, **Supplementary Statistics**). Collectively, these findings demonstrate that combining RTx, oHSV, and IGF1R inhibition synergistically enhances antitumor efficacy in GBM and BC, reduces the effective dose of each agent, and overcomes IGF1R-mediated resistance.

## Discussion

Insulin-like growth factor 1 receptor (IGF1R) signaling has emerged as a critical driver of therapeutic resistance to receptor tyrosine kinase (RTK) inhibitors, chemotherapy, and radiotherapy (RTx) in glioblastoma (GBM) and breast cancer (BC), fueling sustained interest in IGF1R-targeted therapies.^12, 19, 24, 34^ The clinical feasibility of this approach is underscored by the FDA approval of the IGF1R inhibitor teprotumumab (TEPEZZA^®^) in 2020 for thyroid eye disease.^35–37^ However, despite extensive preclinical and clinical investigations, no IGF1R inhibitors have been approved for cancer treatment, likely due to pathways redundancy and compensatory kinase activation.^18^ Our findings suggest that IGF1R remains an exploitable vulnerability when integrated into rationally designed multimodal therapies. Specifically, combining oncolytic herpes simplex virus-1 (oHSV) and RTx, two modalities that converge on IGF1-Rmediated resistance, may enhance therapeutic efficacy and overcome tumor-intrinsic resistance.

Currently, more than 200 clinical trials are evaluating oncolytic viruses (OVs) as monotherapies or in combination regimens, leading to the approval of oHSV talimogene laherparepvec (T-VEC; IMLYGIC^®^, Amgen Inc) for metastatic melanoma.^2, 38, 39^ Despite this progress, the efficacy of oHSV remains limited by both tumor-intrinsic and extrinsic resistance. One major tumor-intrinsic barrier is the compensatory activation of pro-survival pathways downstream of RTKs, such as PI3K/AKT, mTORC1, and MAPK signaling, that promote tumor regrowth. Thus, meticulous investigation of these resistance mechanisms is crucial to optimize next-generation viral constructs and rationally designed combination regimens.^4, 5,40^

We previously demonstrated that oHSV infection induces IGF2 secretion, activating IGF1R signaling and reshaping the tumor immune microenvironment (TIME) by recruiting immunosuppressive myeloid cells (e.g. mMDSCs, gMDSCs).^8^ Herein, we extend these findings by showing that oHSV triggers IGF2/IGF1R activation not only in infected tumor cells but also in neighboring uninfected cells, promoting tumor regrowth and therapeutic resistance. This pro-survival response mirrors the effect of RTx, which similarly upregulates IGF1/IGF1R signaling.^19, 24^ Notably, combining oHSV with RTx further amplified IGF1R activation and downstream YAP1 expression, creating a therapeutic vulnerability that could be exploited through IGF1R inhibition. Indeed, IGF1R blockade suppressed IGF1R signaling and YAP1 expression in a dose-dependent manner, suppressing tumor cell proliferation, self-renewal, and enhancing survival in orthotopic GBM and BC models (Fig. 5C, 5H–I).

YAP and TAZ, key transcriptional co-activators of Hippo signaling, are intimately associated with tumorigenesis, proliferation, stemness, autophagy, recurrence, immunosuppression, and RTx resistance in cancer broadly.^31, 41–44^ Although IGF1R regulation of YAP1 has only recently been implicated in GBM,^45^ the underlying mechanisms remain incompletely defined. In triple-negative breast cancer (TNBC), IGF1/IGF1R/FAK signaling decreases YAP1 phosphorylation, promoting nuclear localization and driving aggressive phenotypes.^46, 47^ Similarly, in diffuse large B-cell lymphoma, IGF1R signaling represses MLK1/2 activity to prevent YAP1 phosphorylation.^48^ Importantly, YAP1 enhances IGF1 and IGF1R expression in both GBM and BC brain metastases (BCBM), creating a feedforward loop driving therapeutic resistance and progression.^46^ This effect was particularly evident in GBM cells harboring the EGFRvIII mutation (Fig. 5C), which exhibit elevated YAP/TAZ activity and heightened sensitivity to YAP/TAZ-targeted therapies.^49^ In BC, YAP1 plays a key role in brain metastasis. Metastatic cells bind astrocyte-derived laminins, which sequester YAP1 and promote dormancy; YAP1 reactivation, triggered by astrocyte relocation (e.g., neuroinflammation or RTx), drives escape from dormancy and accelerated brain colonization.^29, 30, 51–55^ Consistent with this, our analysis of the TCGA BRCA dataset revealed that high YAP1 expression significantly correlated with increased probability of brain metastasis, both in patients with multiple metastatic sites and those with brain-only metastases (**Fig. S15**). These findings suggest that dual RTx and oHSV therapy amplifies YAP1 signaling, potentially enhancing tumor aggressiveness and hindering tumor eradication.

Importantly, the IGF1R-YAP1 signaling axis may reinforce immunosuppression within the TIME, further limiting viro-immunotherapy efficacy. IGF1R and YAP1 signaling promote the recruitment of tumor-associated macrophages (TAMs), which secrete pro-tumorigenic cytokines and reinforce IGF1R/YAP1 activation.^52, 56^ M2-like TAMs and microglia, for example, secrete IGF1, amplifying this feedforward loop^8, 11, 25, 57–59^ In the adaptive immune context, YAP1 promotes the differentiation of regulatory T cells (Tregs)^60^ while suppressing CD8 + cytotoxic T cell infiltration and inducing PD-L1 expression, thereby reducing responses to immune checkpoint blockade (ICB).^56, 61, 62^ Thus, IGF1R/YAP1 signaling may represent a pivotal mechanism underlying both intrinsic and extrinsic resistance to oHSV and RTx.

In conclusion, this study uncovers IGF1R-driven YAP1 expression as a critical adaptation following oHSV and RTx, positioning it as a promising therapeutic target. Disrupting this pathway may enhance tumor control and alleviate immunosuppression, paving the way for rational triple combination strategies. Future studies should delineate how IGF1R/YAP1 modulation reshapes the TIME and determine whether its inhibition can overcome immune evasion to maximize the therapeutic benefit of virotherapy.

## Supplementary Material

Supplementary Files

This is a list of supplementary files associated with this preprint. Click to download.

- SupplementaryStatistics.xlsx
- SupplementalWBProofs.docx
- SupplementFinalizedCDD.docx
- TableS1.pdf
- TableS2.pdf

## Figures and Tables

**Figure 6:**
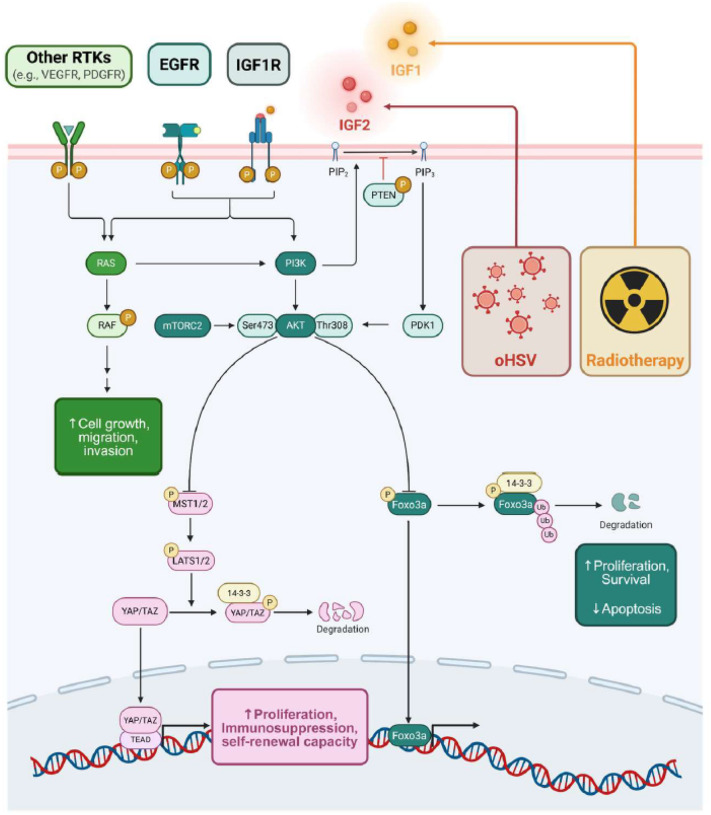
Graphic depicting the proposed mechanism by which combined oHSV and radiotherapy enhance IGF1R signaling and downstream YAP1 expression.

## Data Availability

The data generated in this study are available within the article and its supplementary data files.
